# Applying social life cycle assessment to evaluate the use phase of mobility services: a case study in Berlin

**DOI:** 10.1007/s11367-022-02051-y

**Published:** 2022-04-28

**Authors:** Katharina Gompf, Marzia Traverso, Jörg Hetterich

**Affiliations:** 1 Institute of Sustainability in Civil Engineering, RWTH Aachen University, Aachen, Germany; 2 BMW Group, Knorrstraße 147, 80788 Munich, Germany

**Keywords:** Mobility services, Social life cycle assessment, S-LCA, Use phase, Case study, Social sustainability indicators

## Abstract

**Purpose:**

The main purpose of this S-LCA case study is to analyse social impacts of the use phase of mobility services is a holistic way, taking into account positive as well as negative impacts. The use phase plays an important role for the assessment of services, which is why this study exclusively focuses on the use phase assessment. That way, this study aims to contribute to answer the question whether mobility services can improve quality of life in cities.

**Methods:**

For the analysis, seven different mobility options were chosen in the city of Berlin, Germany, including free-floating car sharing, e-scooter sharing, S-Bahn, subway, tram, bus and the car in private ownership. For the analysis, five stakeholder groups that are outlined in the S-LCA Guidelines (UNEP 2020) were taken into account: Local Community, Consumer, Worker, Value Chain Actors and Society. For a detailed analysis of all relevant aspects, 37 indicators were analysed, out of which 23 are quantitative and 14 are qualitative. For data collection, several different data sources were used, including publicly available data e.g. from statistics as well as own data from interviews.

**Results and discussion:**

For comparability, all results are displayed on a 5-point scale from − 2 to + 2, in line with the Handbook (Goedkoop et al. 2018) and the S-LCA Guidelines (UNEP 2020). For some indicators, the results of the case study are as expected, for example regarding impacts on air quality. For other indicators, however, the results are specific for the analysed mobility services in Berlin and therefore give new insights and reveal new aspects, as for example in the case of job creation for the local community. The main challenge of this S-LCA case study was data availability and data quality, which is why assumptions and simplifications had to be made, especially regarding space occupancy and the allocation of common infrastructure.

**Conclusions:**

This S-LCA case study provides a holistic assessment of the use phase of mobility services, taking into account five stakeholder categories and their respective social impacts. The study illustrates specific results for the city of Berlin, showing positive as well as negative social impacts of mobility services and outlines a procedure for further studies. That way, this case study contributes to answer the overlying question whether mobility services can improve quality of life in cities.

**Supplementary information:**

The online version contains supplementary material available at 10.1007/s11367-022-02051-y.

## Introduction

Urbanisation and rising traffic volumes lead to far reaching problems that influence quality of life in cities around the world. The transportation sector is a major contributor to climate change with 14.3% of the world’s CO_2_ emissions (IPCC 2014). In addition, declining air quality, traffic noise, traffic congestion, fatigue and aggression lead to health impacts, especially in urban areas. On the other hand, cities can also profit from a high population density by more efficient mass transportation (Gross 2019). Moreover, new mobility services are often seen as an opportunity to reduce transport-related impacts in urban areas by reducing private car ownership and offering the possibility to introduce alternatively fuelled transportation options (Gould et al. 2015; Bilali et al. 2020). Mobility services are defined as transportation options that deliver users’ transport needs through an interface of a services provider, which facilitates getting from A to B (Hietanen 2014). Some examples are car sharing, ride hailing, ride pooling, scooter sharing or bike sharing. One major characteristic is that the mobility services can be booked on demand for long- and short-term periods (Jittrapirom et al. 2017).

Different mobility services are rising in numbers, more and more on demand and shared transportation options exist, though the question remains whether different mobility services can contribute to improve quality of life in cities. In order to assess positive as well as negative impacts of different mobility options in a holistic way, it is necessary to assess all three dimensions of sustainability: economic, environmental and social. Life-cycle-based methodologies have been developed over time for a universal assessment (Curran 1996; ISO 2006; Finkbeiner et al. 2010). For the environmental and economic sustainability, a lot of research has been done using life cycle assessment (LCA) and life cycle costing (LCC), respectively. However, a standardised approach for the assessment of social sustainability, using social life cycle assessment (S-LCA), is still missing. Although many questions in S-LCA (theoretical and methodological) still require consensus, great effort has already been made to standardise assessment steps, indicators and database development (Dubois-Iorgulescu et al. 2016; Siebert et al. 2018; Huertas-Valdivia et al. 2020).

An important achievement in the development of S-LCA was the issuing of the UNEP/SETAC S-LCA Guidelines in 2009. According to the UNEP/SETAC Guidelines 2009, “a social and socio-economic Life Cycle Assessment (S-LCA) is a social impact (and potential impact) assessment technique that aims to assess the social and socioeconomic aspects of products and their potential positive and negative impacts along their life cycle” (UNEP/SETAC 2009). In the UNEP/SETAC Guidelines (referred to as the Guidelines hereafter), a framework is given on how S-LCA should be conducted. The framework outlines five stakeholder categories with numerous subcategories, which are characterised with the help of more than 100 inventory indicators. These indicators are published in a separate document: “The methodological sheets for subcategories in social life cycle assessment” (UNEP/SETAC 2013). The indicators given in the methodological sheets are suggestions and the user can choose relevant indicators as well as data sources. After the publication of the Guidelines, many S-LCAs were conducted according to the given framework (Petti et al. 2016; Russo Garrido et al. 2016; Huarachi et al. 2020; Tokede and Traverso 2020). In December 2020, the updated version of the S-LCA Guidelines was published, which includes a sixth stakeholder group, the stakeholder group Children (UNEP 2020). The updated version of the S-LCA Guidelines also integrated Social Organizational LCA (SO-LCA) to assess organisations instead of products and further methodological development reached in the last 10 years has been included.

For the impact assessment, it can generally be differentiated between two main families of impact assessment approaches: the reference scale approach, also referred to as type I impact assessment, and the impact pathway approach, also referred to as type II impact assessment (Parent et al. 2010; Chhipi-Shrestha et al. 2014; UNEP 2020). The reference scale approach uses performance reference points (PRPs) which are defined as thresholds or targets that set different levels of social performance or social risk. The PRPs are context-dependent and are often based on international standards, local legislation or industry best practices (UNEP 2020). Colour-coding, scoring and weighting systems are used for aggregating the inventory indicator data to impact categories (e.g. human rights). The Impact Pathway Approach assesses potential or actual social impacts using cause-effect chains (Weidema 2018). Here, the analysis focuses on identifying and tracking the consequences of activities possibly to longer-term implications along an impact pathway. Midpoint indicators and/or endpoint indicators are used, comparable to LCA (UNEP 2020).

The Handbook for Product Social Impact Assessment (PSIA) is another important milestone. The document was first published in 2014 and has been updated regularly. Mainly developed by industry leaders, it was developed to match the scientific literature with the company strategy. The PSIA handbook also includes some of the stakeholder groups (categories) presented in UNEP (2020). For every stakeholder group, social topics with performance indicators are defined. Work-life balance is for example a social topic and the number of working hours could be the performance indicator to measure the social topic. A qualitative approach is however preferred in the PSIA Handbook.

The PSIA handbook proposes a 5-point scale ranging from − 2 to + 2. This allows to measure positive as well as negative impacts (Fontes et al. 2016; Goedkoop et al. 2018; UNEP 2020).

A variety of S-LCA case studies using different kinds of social indicators and impact assessment methods have been conducted (Di Cesare et al. 2018; Sureau et al. 2019; Tokede and Traverso 2020). Although the number of S-LCA case studies increased massively since 2018, the use phase has been underrepresented (Petti et al. 2016; Kühnen and Hahn 2017; Huarachi et al. 2020; Huertas-Valdivia et al. 2020; Tokede and Traverso 2020). Especially Huertas-Valdivia et al. (2020) as well as Tokede and Traverso (2020) analysed the considered stakeholder groups and subcategories that were included in recent S-LCA case studies. The findings reveal that the stakeholder group Worker received the most attention, while the stakeholder groups Consumer and Local Community have not received as much research attention until now, presumably due to the difficulty in assessing the use phase. However, the use phase plays an important role for the assessment of services in general, also for the assessment of mobility services. Therefore, this study exclusively focuses on the use phase assessment.

The aim of this research is to assess the social impact of the selected mobility services (free-floating car sharing and e-scooter sharing) and compare the impacts to traditional transportation options (S-Bahn, subway, tram, bus and car in private ownership). Thereby, social impacts of the use phase of mobility services are analysed in a holistic way. Thus, this study supports researchers and practitioners in the field of urban mobility assessment as it contributes to answer the question whether mobility services can improve quality of life in urban areas.

## Methods

### Goal, scope and system boundaries

Applying S-LCA to mobility services is a new field of research and no standardised set of social indicators and assessment method exists so far. Gompf et al. (2020) proposed for the first time a holistic set of indicators for the application of S-LCA to mobility services. The suggested indicators are organised in the five stakeholder groups as presented in the Guidelines for S-LCA (UNEP/SETAC 2009): Local Community, User, Worker, Value Chain Actors and Society. As previously mentioned, the updated version of the S-LCA Guidelines includes a sixth group, the stakeholder group Children (UNEP 2020). However, children are not allowed to drive cars and are only indirectly affected by mobility services such as car sharing. In addition, children are also part of the local community and the effects on children are nevertheless included as part of the stakeholder group Local Community. This is why the stakeholder group Children is not separately included in this study that is focusing on the use phase assessment of mobility services.

To answer the question whether mobility services can improve quality of life in cities, the set of indicators and corresponding assessment methods as proposed by Gompf et al. (2020) is applied to selected mobility services and transportation options in the city of Berlin, Germany. For the analysis, free-floating car sharing was selected using the company ShareNow as example. For comparison, the car in private ownership was also analysed. To do so, cars in private ownership that were produced by the BMW Group were used. Further, the standing electric scooter sharing service, (referred to as e-scooter sharing hereafter), with the companies Tier and Voi, were selected. In order to be able to compare these mobility services to existing public transportation options, the S-Bahn Berlin, operated by the German railway company Deutsche Bahn, and subway, tram and bus, operated by BVG (Berlin public transportation company), were also included in the analysis.

**Fig. 1:**
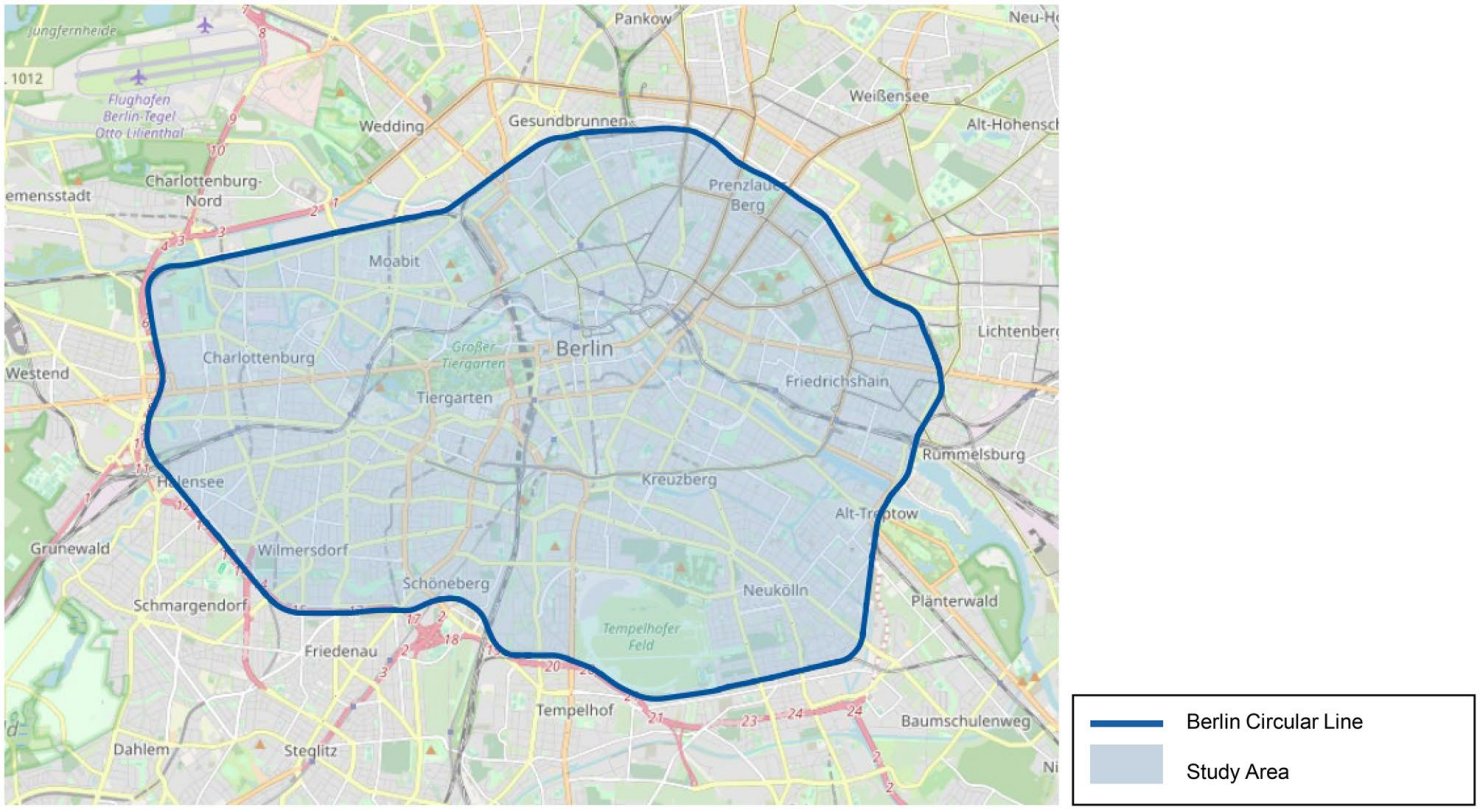
Geographic system boundary and study area

For the analysis of the various impacts, data was collected in the city of Berlin for free-floating car sharing, e-scooter sharing, S-Bahn, subway, tram, bus and the car in private ownership using the chosen set of indicators by Gompf et al. (2020). As geographic system boundary, the circular line of the Berlin S-Bahn is used, as the area within the circular line corresponds to the operating area of the different transportations options under analysis and represents the inner city of Berlin. The geographic system boundary is illustrated in Fig. 1.

The analysed stakeholder categories, corresponding subcategories and indicators, type of indicator, calculation formula and performance indicators can be seen in Table 1. For the selection of the subcategories and indicators, Gompf et al. (2020) did an extensive literature review based on defined search strings in the field of S-LCA and mobility services. Fifty-one publications were identified, including 579 social indicators. The analysis revealed a wide variety and diversity of indicators that are trying to measure the same aspect. The indicators were allocated to the respective stakeholder groups and selected based on the relevance for mobility services. This led to a core set of 39 indicators. For a weighting of the indicators, Gompf et al. (2021) used the analytical hierarchy process (AHP) method using expert judgements in a participatory approach. However, direct stakeholders’ point of view is not included in the selection of the indicators, which leaves the research limited to literature review and experts’ point of view, as rightly pointed out by do Carmo et al. (2021). For the indicators analysing local job creation, the number of consumer complaints as well as the number of audited suppliers, it was not possible to collect all necessary data. This is why these three indicators were excluded from the analysis. This leads to 36 indicators that were analysed, out of which 22 are quantitative and 14 are qualitative.

**Table 1:** Overview of analysed indicators, necessary data, data sources and used reference scales

**Category**	**Indicator**	**Type of indicator**	**Calculation formula and description**	**Necessary data**	**Data sources**	**Reference scale**
Local community						
Public space						
	Green and open space per capita	Quantitative	$Green\;and\;open\;space=\frac{Park\;green\;are\;and\;oprn\;space\;\lbrack m^2\rbrack}{Number\;of\;inhabi\tan ts}$	Area of parks, area of public space, number of inhabitants	QGIS/open source	See Table 7 in Gompf et al. (2020)
Air quality						
	Emission intensity of NOx	Quantitative	$Emission\;intesity\;No_x=\frac{No_x\;\left[mg\right]}{Passenger\;kilometre}$	Emissions of NOx per passenger km for all transport modes	HBEFA/LCA data	See Table 7 in Gompf et al. (2020)
	Emission intensity of PM10	Quantitative	$Emission\;intesity\;PM10=\frac{10\;\left[mg\right]}{Passenger\;kilometre}$	Emissions of PM10 per passenger km for all transport modes	HBEFA/LCA data	See Table 7 in Gompf et al. (2020)
	Emission intensity of PM2.5	Quantitative	$Emission\;intesity\;PM2.5=\frac{2.5\;\left[mg\right]}{Passenger\;kilometre}$	Emissions of PM2.5 per passenger km for all transport modes	HBEFA/LCA data	See Table 7 in Gompf et al. (2020)
	Emission intensity of SO_2_	Quantitative	$Emission\;intesity\;SO_2=\frac{SO_2\;\left[mg\right]}{Passenger\;kilometre}$	Emissions of SO_2_ per passenger km for all transport modes	HBEFA/LCA data	See Table 7 in Gompf et al. (2020)
Employment						
	Percentage of employees hired	Quantitative	$Job\;creation=\frac{Number\;of\;employees\;hired}{Total\;number\;of\;employees}\times100$	Hired employees, total number of employees at country level	Interview/open source	See Table 7 in Gompf et al. (2020)
	Percentage of employees hired locally	Quantitative	$\mathrm{Local}\;\mathrm{job}\;\mathrm{creation}=\frac{Number\;of\;employees\;hired\;locally}{Total\;number\;of\;employees}\times100$	Locally hired employees, total number of employees at city evel	Interview/open source	See Table 7 in Gompf et al. (2020)
Noise pollution						
	Noise pollution greater than 65 dB	Quantitative	$\mathrm{Noise}\;\mathrm{pollution}=\frac{Inhabited\;area\;with\;noise\;pollution>65dB\;in\;m^2}{total\;study\;area\;m^2}\times100$	Area exceeding noise level of 65 dB, study area [m^2^]	QGIS/open source	See Table 7 in Gompf et al. (2020)
	Average emissions of noise	Quantitative	$$\begin{array}{l}\mathrm{Noise}\;\mathrm{index}=\;\frac{Area>62dB\;in\;cm^2\;\times\;MWF}{MWF\;\lbrack study\;area\rbrack\;\times Passenger\;kilometre} \\ \mathrm{MWF}=\;\mathrm{Measurement}\;\mathrm{Weight}\;\mathrm{Factor}\;(\mathrm{depending}\;\mathrm{on}\;\mathrm{population}\;\mathrm{density}\;\mathrm{of}\;\mathrm{the}\;\mathrm{area},\;\mathrm{considering}\;\mathrm{twelve}\;\mathrm{density}\;\mathrm{classes})\\ \mathrm{MWF}\;\lbrack\mathrm{study}\;\mathrm{area}\rbrack=\mathrm{MWF}\;\mathrm{according}\;\mathrm{to}\;\mathrm{inhabitants}/\mathrm{ha}\;\mathrm{in}\;\mathrm{study}\;\mathrm{area}\;\end{array}$$	Area exceeding noise level of 65 dB, dwellings in study area	QGIS/open source	See Table 7 in Gompf et al. (2020)
Community engagement						
	Degree of population participation	Qualitative	The extent to which the company or facility engages with community stakeholders through ongoing open dialogue and responds to their concerns and inquiries fairly and promptly, to continuously foster greater trust and relationship with the local community.	Company’s engagement with residents	Interview/open source	See Goedkoop et al. (2018), p. 58
Space occupancy						
	Infrastructure efficiency	Quantitative	$Infrastructure\;efficiency=\;\frac{Direct+Indirect\;space\;for\;mobility\;mode\;i\;\lbrack m^2\rbrack}{Passenger\;kilometre}$	Occupied space per mobility mode, passenger km	QGIS/open source	See Table 7 in Gompf et al. (2020)
	Infrastructure space occupancy	Quantitative	$Infrastructure\;space\;occupancy=\;\frac{Direct+Indirect\;space\;for\;mobility\;mode\;i\;\lbrack m^2\rbrack}{Total\;study\;area\;\lbrack m^2\rbrack}$	Occupied space per mobility mode, study area	QGIS/open source	See Table 7 in Gompf et al. (2020)
	Space occupancy in relation to green and open space	Quantitative	$Space\;occupancy=\;\frac{Space\;occupancy\;\lbrack m^2\rbrack}{Park\;green\;area\;and\;open\;space\;\lbrack m^2\rbrack}$	Occupied space per mobility mode, public space	QGIS/open source	See Table 7 in Gompf et al. (2020)
Consumers						
Accessibility						
	Number of transport points	Quantitative	$\mathrm{Accessibility}\;\lbrack1\rbrack=\frac{Total\;number\;of\;transport\;points}{study\;area\;\lbrack km^2\rbrack}$	Numbers of public transport stations, number of free-floating vehicles in study area	QGIS/open source	See Table 7 in Gompf et al. (2020)
	Number of passengers	Quantitative	Accessibility [2] = total number of passengers per mobility mode	Passengers per mobility mode	QGIS/open source	See Table 7 in Gompf et al. (2020)
Safety						
	Fatal and non-fatal traffic accidents	Quantitative	$\mathrm{Safety}=\frac{Number\;of\;fatal\;and\;non-fatal\;accidents}{passenger\;kilometre}$	Accidents per mobility mode	Interview/open source	See Table 7 in Gompf et al. (2020)
Convenience						
	Punctuality of deliveries	Quantitative	$\mathrm{Punctuality}=\;\frac{Number\;of\;puntual\;trips\;with\;3\;minutes\;tolerance}{Total\;number\;of\;trips}$	Punctuality statistics for each transport mode	Open source	See Table 7 in Gompf et al. (2020)
Inclusiveness						
	Inclusive design (ageing and disabled)	Qualitative	Measures the extent to which a product design, marketing and company business models affect the affordability and accessibility of products or services to different groups of people, e.g. disabled persons, the elderly and persons with low income.	Company’s efforts for an inclusive service	Interview/open source	See Goedkoop et al. (2018), p. 53
Affordability						
	Trip fare	Quantitative	$\mathrm{Affordability}=\frac{Fare\;of\;5km\;trip\;within\;study\;are}{Average\;income}$	Trip fare in study area, average income in Berlin	Open source	See Table 7 in Gompf et al. (2020)
Privacy						
	Data privacy	Qualitative	Measures the extent to which a company respects and protects users` data privacy.	Privacy policy of transport company	Interview/open source	See Goedkoop et al. (2018), p. 51
Feedback mechanism						
	Consumer complaints	Quantitative	$\mathrm{Consumer}\;\mathrm{satisfaction}=\;\frac{Total\;number\;of\;complaints}{passenger\;kilometre}$	Statistics for each mobility mode	Interview/open source	See Table 7 in Gompf et al. (2020)
Worker						
Safety						
	Fatal and non-fatal injuries	Quantitative	$\mathrm{Safety}=\frac{Number\;of\;fatal\;and\;non-fatal\;injuries}{passenger\;kilometre}$	Number of employees, number of accidents within company	Interview/open source	See Table 7 in Gompf et al. (2020)
Fair salary						
	Remuneration	Qualitative	The extent to which management compensates workers. This indicator measures a combination of wages and social benefits received by workers.	Average wage, social benefits	Interview	See Goedkoop et al. (2018), p. 42
	Minimum wage paid	Quantitative	$\mathrm{Minimum}\;\mathrm{wage}\;\mathrm{paid}=\;\frac{Number\;of\;workers\;with\;at\;least\;minimum\;wage}{Total\;number\;of\;employees}\times100$	Number of workers with at least minimum wage	Interview	See Table 7 in Gompf et al. (2020)
Discrimination						
	Prevention of discrimination	Qualitative	Measures the extent to which a company is engaged in preventing discrimination and pro-actively promoting non-discrimination at the workplace. Discrimination refers to any distinction, exclusion or preference which has the effect of nullifying or impairing equality of opportunity or treatment.	Code of conduct, non-discrimination policy	Interview/open source	See Goedkoop et al. (2018), p. 45
Child labour						
	Prevention of child labour	Qualitative	Measures the extent to which a company works towards eradicating child labour and pro-actively raising awareness of issues associated with child labour.	Code of conduct, company’s policy	Interview/open source	See Goedkoop et al. (2018), p. 43
Freedom of association and collective bargaining						
	Freedom of association and collective bargaining	Qualitative	Measures the extent to which workers have the right to establish and to join organisations of their choice without prior authorisation, to promote and defend their interests and to negotiate collectively with other parties.	Code of conduct, company’s policy	Interview	See Goedkoop et al. (2018), p. 46
Work-life balance						
	Healthy work-life balance	Qualitative	Measures the extent to which a company enables workers to have choices over when, where and how they work and encourages healthy work-life balance.	Company’s offerings for workers	Interview	See Goedkoop et al. (2018), p. 47
Forced labour						
	Prevention of forced labour	Qualitative	Measures the extent to which forced labour is occurring and the mechanisms to prevent this.	Code of conduct, company’s policy	Interview/open source	See Goedkoop et al. (2018), p. 44
Value Chain Actors						
Fair competition						
	Fair competitive activities	Qualitative	Measures the extent to which the organisation’s competitive activities are conducted in a fair way and in compliance with legislations preventing anti-competitive behaviour, anti-trust or monopoly practices.	Company’s policy	Interview	See Table 8 in Gompf et al. (2020)
Intellectual property rights						
	Respect of Intellectual Property Rights	Qualitative	Measures the extent to which the organisation’s actions safeguard and value intellectual property rights.	Company’s policy	Interview	See Table 8 in Gompf et al. (2020)
Supplier relationships						
	Purchasing behaviour	Qualitative	Measures the extent to which a company is minimising negative impacts of procurement and purchasing decisions on other organisations.	Company’s policy	Interview	See Table 8 in Gompf et al. (2020)
Promoting social responsibility						
	Social responsibility support	Qualitative	Measures the extent to which a company supports suppliers in terms of consciousness-raising and counselling concerning social responsibility issues.	Company’s policy	Interview/open source	See Table 8 in Gompf et al. (2020)
	Percentage of audited suppliers	Quantitative	$Promoting\;Social\;Responsibility=\;\frac{Number\;of\;audited\;suppliers}{Total\;number\;of\;suppliers}\times100$	Audited suppliers, total number of suppliers	Interview	See Table 7 in Gompf et al. (2020)
Society						
Health						
	GWP100 [CO_2_ equiv.]	Quantitative	$\mathrm{GWP}100=\frac{Co_2\;equiv\;\lbrack g\rbrack}{Passenger\;kilometre}$	Direct and indirect emissions during use-phase	LCA data	See Table 7 in Gompf et al. (2020)
	Acidification potential [SO_2_ equiv.]	Quantitative	$\mathrm{AP}=\frac{SO_2\;equiv\;\lbrack mg\rbrack}{Passenger\;kilometre}$	Direct and indirect emissions during use-phase	LCA data	See Table 7 in Gompf et al. (2020)
	Eutrophication potential [PO_4_ equiv.]	Quantitative	$EP=\frac{PO_4\;equiv\;\lbrack mg\rbrack}{Passenger\;kilometre}$	Direct and indirect emissions during use-phase	LCA data	See Table 7 in Gompf et al. (2020)
Urban development						
	Urban development plans	Qualitative	Measures the extent to which a company is engaging with city authorities to actively contribute to urban development.	Company’s engagement with city of Berlin	Interview	See Table 8 in Gompf et al. (2020), Table 8
Tax income						
	Taxes per pkm	Qualitative	$Tax\;income=\frac{Paid\;taxes\;[\EUR]}{passenger\;kilometre}$	Paid taxes bycompany or rate of value added tax	Interview/open source	See Table 7 in Gompf et al. (2020)

**Passenger kilometer: **The applied passenger kilometres for the calculation were taken from publicly available data with the following values: S-Bahn: 4,800 million pkm (S-Bahn Berlin 2021); subway: 2,651.8 million pkm (Berliner Verkehrsbetriebe (BVG) 2019); bus: 1,523.1 million pkm (Berliner Verkehrsbetriebe (BVG) 2019); tram: 627.7 million pkm (Berliner Verkehrsbetriebe (BVG) 2019); E-scooter: 39,676,555 pkm (Civity 2021); car-sharing: 61,026,120 pkm (ShareNow 2021); car in private ownership: 16,201.9 million pkm (Gerike et al. 2020)

### Social life cycle inventory

For data collection, several different data sources were used. First, publicly available data was used where possible. Among the publicly available data, the main source was QGIS, an open source Geographic Information System. QGIS not only includes a detailed map of Berlin but also detailed information about public transportation, traffic noise or population density. In addition to QGIS, publicly available company reports were used, as for example the sustainability reports of the selected companies or their code of conduct. Second, the social hotspot database (Benoit-Norris et al. 2012) was used as well as LCA data from the GaBi software. LCA data was necessary for selected indicators in the categories air quality and health, evaluating emission intensities per passenger kilometre. In case of a combined assessment of environmental and social impacts, double counting should be avoided for these indicators. Third, for all qualitative indicators or indicators with no publicly available data, a questionnaire was developed. With the questionnaire, selected employees in the field of sustainable urban mobility were questioned from the companies ShareNow (free-floating car sharing), Voi and Tier (e-scooter sharing), the German railway company Deutsche Bahn (S-Bahn Berlin) and the Berlin public transportation company BVG (subway, bus and tram) as well as the company BMW Group (representation of cars in private ownership). In total, 15 interviews were conducted with at least two selected experts representing each company.

For the selection of the interviewed persons, work experience and expertise in sustainable mobility was a requirement as well as thorough knowledge of the company in question (e.g. higher management position). With the developed questionnaire, guided interviews were conducted, using a script. (For an overview of the necessary data and used data sources, see Table 1). The developed questionnaire can be found in the supplementary materials. In Table 2, an example of the inventory data for free floating car sharing is presented for all quantitative indicators of the stakeholder group Local Community. An overview of all inventory data can also be found in the supplementary materials.

**Table 2:** Inventory data for the stakeholder group Local Community and free-floating car sharing

				**Free-floating car sharing**
**Category**	**Indicator short name**	**Unit**	**Indicator value**	**Reference value**
Local Community				
Green and open space per capita	Green and open space per capita	$\frac{[{\mathrm{m}}^{2}]}{\mathrm{Inhabitants}}$	11.67	0
Emission intensity of NOx	NOx	$\frac{NOx \left[mg\right]}{Pkm}$	398.67	0
Emission intensity of PM10	PM10	$\frac{PM10 \left[mg\right]}{Pkm}$	14.98	0
Emission intensity of PM2.5	PM2.5	$\frac{PM2.5 \left[mg\right]}{Pkm}$	15.31	− 1
Emission intensity of SO_2_	SO_2_	$\frac{SO2 \left[mg\right]}{Pkm}$	0.21	0
Percentage of employees hired	Job creation	[%]	− 11	− 2
Noise pollution greater than 65 dB	Noise pollution	[%]	0.08	1
Average emissions of noise	Noise index	$\frac{\left[c{m}^{2}\right] }{Pkm}$	10.89	− 1
Infrastructure efficiency	Infrastructure efficiency	$\frac{\left[{m}^{2}\right] }{Pkm}$	0.23	2
Infrastructure space occupancy	Infrastructure space occupancy	[%]	0.02	2
Space occupancy in relation to green and open space	Space occupancy	[%]	0.15	1

### Social life cycle impact assessment

For the impact assessment, the reference scale approach (type I) was used. As already mentioned, the PSIA handbook as well as the Guidelines (UNEP 2020) proposes a 5-point scale ranging from − 2 to + 2 to be able to assess positive as well as negative impacts. In general, the score 0 is used for neutral performance, compliant with local and international laws and/or basic societal expectations. A score of − 1 is slightly below compliance level or slightly below average performance, whereas − 2 is used for starkly below compliance level or starkly below average performance. Consequently, a result of + 1 is used for beyond compliance or slightly above average performance and + 2 is used for ideal performance or best in class. In order to be able to assess the collected data against the proposed 5-point scale, performance reference points (PRPs) need to be defined for every level on the scale and a reference scale with PRPs is necessary for every indicator.

For this study, the reference scales with the defined PRPs by Gompf et al. (2020) were adopted for all quantitative indicators. These reference scales were developed using possible minimum and maximum values for the transportation options under analysis (free-floating car sharing, e-scooter sharing, S-Bahn, subway, tram, bus and the car in private ownership). The definition of the PRPs was done as described by the Guidelines (UNEP 2020). Each scale level was granted a customised value according to its judged distance with the other scale levels. For example, the indicator analysing PM_10_ emissions is measuring the emission intensity of PM_10_ per passenger kilometre (pkm). After data collection for the different transportation options under analysis, a range of values between 0.24 mg PM_10_ per passenger kilometre (S-Bahn) and 21.18 mg PM_10_ per passenger kilometre (bus) is revealed. For a reasonable reference scale that allows to classify all transportation options, the range between the minimum and the maximum score was divided in five equal parts, leading to linear scores. In this example, 0–5 mg/pkm is resulting in a + 2, 5–10 mg/pkm is resulting in a + 1, 10–15 mg/pkm leads to an average score of 0, 15–20 mg/pkm is resulting in a − 1 and every value above 20 mg/pkm leads to a score of − 2. In the same way, the reference scales were developed for all quantitative indicators.

For most of the qualitative indicators, the reference scales and PRPs as proposed by Goedkoop et al. (2018) were adopted. (For an overview of the used reference scales and defined PRPs for all indicators, see also Table 1.) Whenever possible, passenger kilometre was used as functional unit. However, especially for the qualitative indicators, this was not practical, which is why there is no consistent functional unit for all indicators. In Table 3, the adopted reference scales and PRPs from Gompf et al. (2020) can be found for all quantitative indicators. Assessing the inventory indicator data against the PRPs allows to classify the collected data on the 5-point scale. In Table 2, not only the indicator values can be found but also the reference values after assessing the data against the PRPs. If we take for example the first indicator of the stakeholder group Local Community, green and open space per capita in m^2^, the indicator value is 11.67m^2^ per inhabitant. If we assess this value against the PRPs, we can see that the value lies between 10 and 25 m^2^ per inhabitant. This leads to a score of 0 on the reference scale. This is done for every indicator of every stakeholder group and for every transport mode (free-floating car sharing, e-scooter sharing, S-Bahn, subway, tram, bus and the car in private ownership) to get the corresponding reference values.

**Table 3:** Reference scale and performance reference points for all quantitative indicators, adopted from Gompf et al. (2020)

**Category**	**Indicator**	**Unit**	**Reference scale and performance reference points**				
			** − 2**	** − 1**	**0**	**1**	**2**
Local Community							
Public space							
	Green and open space per capita	$\frac{\lbrack m^2\rbrack}{inhabitants}$	0–5	5–10	10–25	25–50	> 50
Air quality							
	Emission intensity of NOx	$\frac{NO_x\;\lbrack mg\rbrack}{Pkm}$	> 1000	475–1000	50–475	1–50	0–1
	Emission intensity of PM10	$\frac{PM10\;\lbrack mg\rbrack}{Pkm}$	> 20	15–20	10–15	5–10	0–5
	Emission intensity of PM2.5	$\frac{PM2.5\;\lbrack mg\rbrack}{Pkm}$	> 20	15–20	10–15	5–10	0–5
	Emission intensity of SO_2_	$\frac{SO_2\;\lbrack mg\rbrack}{Pkm}$	> 1	0.75–1	0.5–0.75	0.25–0.5	0–0.25
Employment							
	Percentage of employees hired	[%]	> − 10	− 10 to − 5	− 5 to + 5	5–10	< 10
Noise pollution							
	Noise pollution greater than 65 dB	[%]	> 20	10–20	1–10	0.1–1	0
	Average emissions of noise	$\frac{\lbrack cm^2\rbrack}{Pkm}$	> 20	10–20	1–10	0.1–1	0
Space occupancy							
	Infrastructure efficiency	$\frac{\lbrack m^2\rbrack}{Pkm}\ast$	> 1	0.75–1	0.5–0.75	0.25–0.5	0–0.25
	Infrastructure space occupancy	[%]	> 50	10–50	1–10	0.1–1	0–0.1
	Space occupancy in relation to green and open space	[%]	> 50	10–50	1–10	0.1–1	0–0.1
Consumers							
Accessibility							
	Number of transport points	Count	< 50	50–100	100–500	500–1000	> 1000
	Number of passengers	Count (in millions)	< 10	10–100	100–250	250–500	> 500
Safety							
	Fatal and non-fatal traffic accidents	Accidents/million pkm	> 2.0	1.0–2.0	0.5–1.0	0.1–0.5	0–0.1
Convenience							
	Punctuality of deliveries	[%]	< 80	80–90	90–95	95–99	100
Affordability							
	Trip fare	€	> 4.5	3.5–4.5	2.5–3.5	1.5–2.5	0–1.5
Worker							
Safety							
	Fatal and non-fatal injuries	Injuries/1000 employees	> 60	45–60	30–45	15–30	0–15
Fair salary							
	Minimum wage paid	[%]	> 80	80–90	90–95	95–99	100
Value Chain Actors							
Promoting social responsibility							
	Percentage of audited suppliers	[%]	0–20	20–40	40–60	60–80	80–100
Society							
Health							
	GWP100 [CO_2_ equiv.]	[g/pkm]	> 160	120–160	80–120	40–80	0–40
	Acidification potential [SO_2_ equiv.]	[mg/pkm]	> 400	300–400	200–300	100–200	0–100
	Eutrophication potential [PO_4_ equiv.]	[mg/pkm]	> 100	75–100	50–75	25–50	0–25
Tax income							
	Taxes per pkm	[€/pkm]	0–0.02	0.02–0.04	0.04–0.06	0.06–0.08	> 0.08

*Indicator values × 1000 for reference scale

The procedure for all qualitative indicators is the same. The only difference is that the inventory data is based on the conducted interviews and the PRPs as defined by Goedkoop et al. (2018) are used to derive the reference values. All reference values can also be found in the supplementary materials.

## Social life cycle impact assessment results

**Fig. 2:**
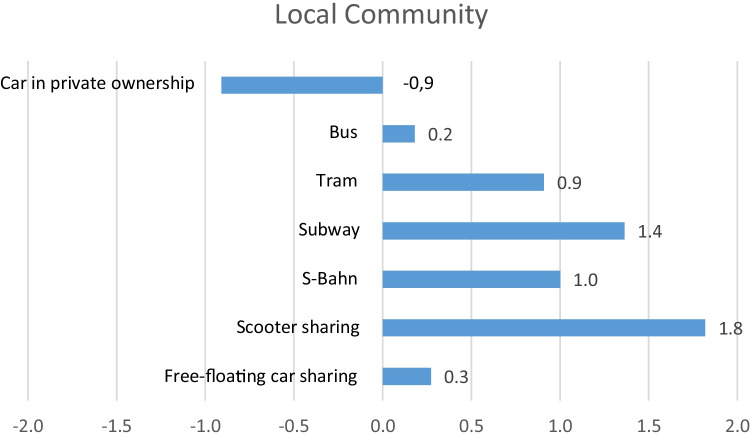
Aggregated results for the stakeholder group Local Community

**Fig. 3:**
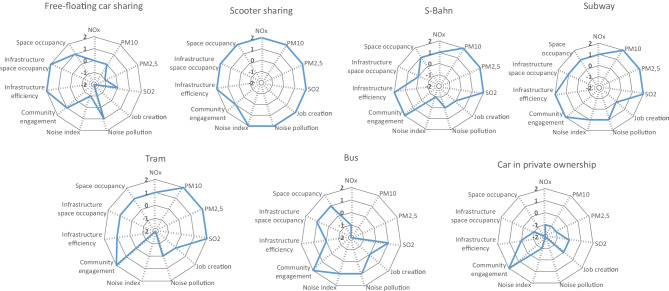
Detailed results for the stakeholder group Local Community

After assessing all the collected data against the adopted reference scales and PRPs, the results are presented as one concise aggregated graph for each stakeholder group as well as separate radar charts for each mobility service. For the aggregated graphical presentation, the results for the single indicators within one mobility service are all added up and then divided by the number of indicators for each mobility service. That way, the average score can be presented for a better overview and easier analysis of trade-offs. In addition, for full insights and a detailed presentation of the results, separate radar charts for each mobility service are provided. In Fig. 2, the aggregated results for the stakeholder group Local Community can be seen, and in Fig. 3, the detailed results are available as separate radar charts for each mobility service. In this stakeholder category, the indicator evaluating community engagement is the only qualitative indicator. All other indicators are quantitative. When examining the radar charts with the results for the different mobility services, it is obvious that the air quality indicators (NOx, PM10, PM2.5, SO_2_) reveal average or below average performance for free-floating car sharing and the car in private ownership as well as the bus. Especially the bus shows clearly below average results for PM10 and PM2.5. Although the results are calculated per passenger kilometre and an average capacity utilisation rate is applied for the calculation, the bus still receives worse scores than the car. This is because the average capacity utilisation rate for buses is comparably low, mostly due to outside rush hour times.

Contrary, the e-scooter sharing service, the S-Bahn, subway and tram reveal best scores with + 1 for NOx and + 2 for PM10, PM2.5 and SO2. Regarding job creation, free-floating car sharing receives a score of − 2. E-scooter sharing, however, receives a score of + 2, whereas all other transport options receive an average score (0). The indicator measuring noise pollution is analysing the inhabited area in m^2^ that is affected with a noise pollution above 65 dB. For this indicator, free-floating car sharing receives a score of + 1, just like subway and bus. S-Bahn and tram receive an average score (0), whereas the car in private ownership reveals a score of − 2. Only the e-scooter sharing service receives the best possible score (+ 2). The indicator noise index does not only consider the area that is affected by noise but also the population that is affected by noise in the study area by also taking into account the population density (number of inhabitants per km^2^). Considering this for the evaluation of noise emissions, free-floating car sharing receives a score of − 1, just like S-Bahn and the car in private ownership. Subway and bus both receive a score of + 1, whereas the tram shows the worst score for this indicator with − 2. Only the e-scooter sharing service receives again the best possible score (+ 2). Having a detailed look at community engagement as the only qualitative indicator in this stakeholder group, almost all analysed services reveal above average results. Free-floating car sharing receives a + 1, just like the e-scooter sharing service. S-Bahn, subway, tram, bus and the car in private ownership all receive a score of + 2.

The space occupancy indicators (infrastructure efficiency, infrastructure occupancy and space occupancy) analyse three different perspectives of the same aspect. Infrastructure efficiency measures the space that is occupied per transport mode in relation to passenger kilometres. Infrastructure occupancy measures the space that is occupied per transport mode in relation to green and open space, while the space occupancy indicator simply measures the space that is occupied per transport mode in the study area. As these indicators measure the same aspect from different perspectives, the results for all three indicators are similar for the analysed mobility services. The e-scooter sharing service performs best with + 2 for all three indicators. This is followed by free-floating car sharing (+ 2, + 2, + 1). The rail-bound services (S-Bahn, subway, tram) show average or above average results between 0 and + 2. The bus reveals average or slightly above average results (0, + 1, + 1) for the space occupancy indicators. Finally, the car in private ownership discloses the worst scores in this category (0, − 1, − 2). The indicator evaluating green and open space per capita is also included in the list of indicators for the stakeholder group Local Community. This indicator reveals a medium score for the city of Berlin (0), though this is not included in the radar charts as it is identical for all transportation options. Nevertheless, this indicator is essential as mobility services, just like other transportation systems, require infrastructure, which may lead to green space destruction. At the same time, higher occupancy rates or efficiency may also lead to less space occupancy and, consequently, to new possibilities for green or open space. Therefore, an indicator evaluating these aspects is highly relevant and nevertheless included in the study.

Generally speaking, the results for the stakeholder group Local Community are best for the e-scooter sharing service and the subway. Especially regarding the air quality indicators, not only the e-scooters and the subway but also all rail-bound services show advantages. Except for community engagement, all mobility services reveal better results than the car in private ownership.

**Fig. 4:**
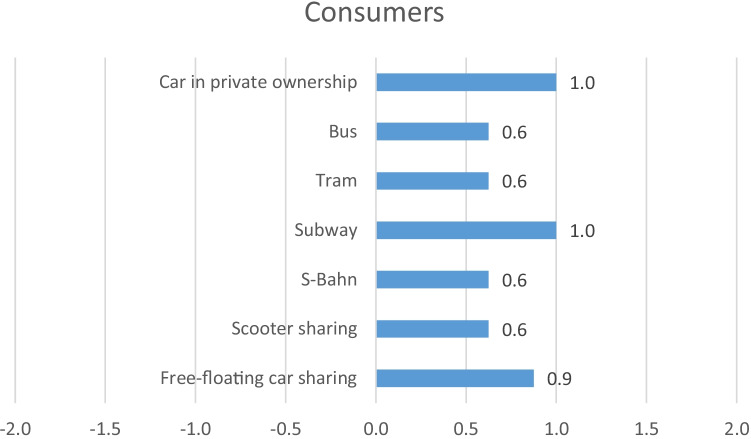
Aggregated results for the stakeholder group Consumer

**Fig. 5:**
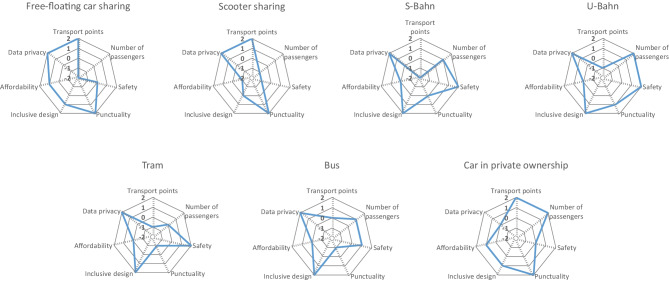
Detailed results for the stakeholder group Consumer

In Fig. 4, the aggregated results for the stakeholder group Consumer are presented, and in Fig. 5, the detailed results can be found as separate radar charts. When analysing free-floating car sharing, it is noticeable that the two indicators measuring accessibility (number of transport points and number of passengers) reveal opposing results (+ 2 and − 2). This reflects the fact that within the study area there are many transport points, in this case car sharing cars; however, the total number of passengers using car sharing is comparatively very low, which leads to a clearly below average result. For the analysed e-scooter sharing service, the results are similar. Again, many sharing e-scooters are available within the study area, though the total number of passengers is comparably low. All other mobility services show opposing results, as there are comparably few transport points (S-Bahn, subway, tram or bus stations), however, a lot of passengers. Only the car in private ownership shows very good scores for both indicators, as it is assumed the own car itself is counted as transport point.

When analysing consumer safety, measured as fatal and non-fatal accidents per passenger kilometre, free-floating car sharing as well as the car in private ownership shows average results (0), whereas the e-scooter sharing service reveals the worst score for this indicator (− 1). Contrary to the e-scooter sharing service, S-Bahn, subway and tram show the best scores with + 2, each. The bus reveals a slightly above average score of + 1. Having a look at the punctuality of the different mobility services, free-floating car sharing as well as e-scooter sharing and the car in private ownership receive best possible scores (+ 2), as it is assumed that there is no waiting time for these transportation options. The S-Bahn receives an average score (0), whereas the subway receives a slightly above average score (+ 1). Tram and bus, however, receive slightly below average scores with − 1, each. The indicator inclusive design evaluates to what extent the different mobility services affect the affordability and accessibility of different groups of people, e.g. disabled persons, the elderly or persons with low income. This indicator is the only qualitative indicator of the stakeholder group Consumer. Whereas free-floating car sharing receives a score of + 1, the e-scooter sharing service only receive an average score (0). S-Bahn, subway, tram and bus, however, each reveal scores of + 2. The car in private ownership is evaluated with a score of + 1. The indicator evaluating affordability of different transport modes measures the trip faire for a distance of 5 km within the study area in relation to average income. The results demonstrate that of all analysed mobility services, the e-scooter sharing service is the most expensive (− 1). Free-floating car sharing and the car in private ownership both receive a score of + 1, whereas S-Bahn, subway, tram and bus all receive the average score of 0. Having a look at data privacy, which measures the extent to which the respective company protects user’s data, free-floating car sharing, e-scooter sharing and S-Bahn, subway, tram and bus all receive a score of + 2. For all these services, the EU data privacy regulation applies, which explains the positive scores. For the car in private ownership, however, the evaluation reveals an average score, as especially in new cars a lot of data is collected, often with a lack of transparency for the driver. When evaluating consumer complaints, free-floating car sharing and the tram receive a score of + 1, whereas e-scooter sharing reveals a score of + 2. S-Bahn, subway and the bus receive an average score (0), just like the car in private ownership.

In an overall comparison of the single indicators of the stakeholder group Consumer, none of the analysed mobility services reveals a clear advantage over other services. Every mobility service shows very good results for some indicators, however medium or poor results for other indicators. Only the car in private ownership does not reveal any below average results.

**Fig. 6:**
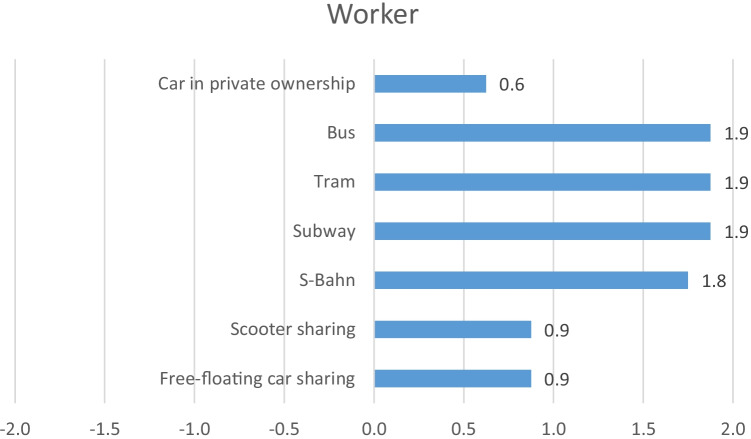
Aggregated results for the stakeholder group Worker

**Fig. 7:**
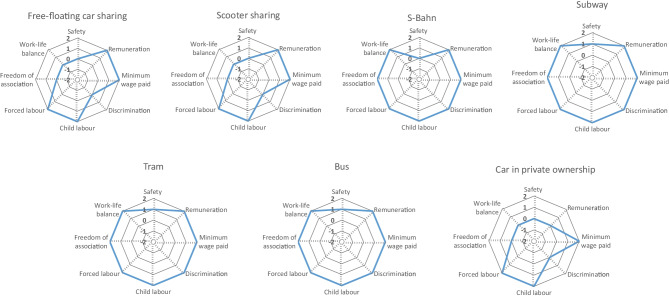
Detailed results for the stakeholder group Worker

In Fig. 6, the aggregated results for the stakeholder group Worker are presented, and in Fig. 7, the detailed results can be found again as separate radar charts for each mobility service. Unlike the stakeholder group Local Community or Consumer, most of the indicators in the stakeholder group Worker are qualitative. As the focus of this study is on the use phase, it should be highlighted at this point that the stakeholder group Worker represents in this case worker related to the use phase, not worker during production of the respective mobility service. This means for example a bus or tram driver or a car sharing or e-scooter sharing fleet manager. In the case of the car in private ownership, workers at service stations are chosen as representation of workers in the use phase of the car.

The only two quantitative indicators in this stakeholder group are safety, evaluating the number of fatal or non-fatal accidents, and minimum wage paid. Having a closer look at worker’s safety, it can be seen that free-floating car sharing and e-scooter sharing receive the worst scores with − 1. The S-Bahn as well as the car in private ownership receives medium scores, whereas subway, tram and bus receive a score of + 1. Regarding remuneration, all mobility services except the car in private ownership receive the highest score (+ 2). For all analysed mobility services, regulations and corresponding policies regarding minimum wage are in place and no violation of minimum wage was detected, which is why all services receive a + 2. For free-floating car sharing and e-scooter sharing as well as for the car in private ownership, prevention of discrimination obtains average scores. All the other mobility services, however, receive the highest score with + 2. Concerning prevention of child labour and prevention of forced labour, likewise corresponding regulations and policies are in place and no violation was detected, which is why for those two aspects all mobility services and the car in private ownership receive a + 2. Concerning freedom of association as well as work-life balance, free-floating car sharing, e-scooter sharing and the car in private ownership receive average scores, whereas S-Bahn, subway, tram and bus reveal best possible scores (+ 2).

Overall, it can be stated that the public transportation services (S-Bahn, subway, tram and bus) reveal the best results regarding the stakeholder group Worker, whereas free-floating car sharing and e-scooter sharing as well as the car in private ownership reveal improvement potential especially concerning freedom of association, work-life balance and safety.

**Fig. 8:**
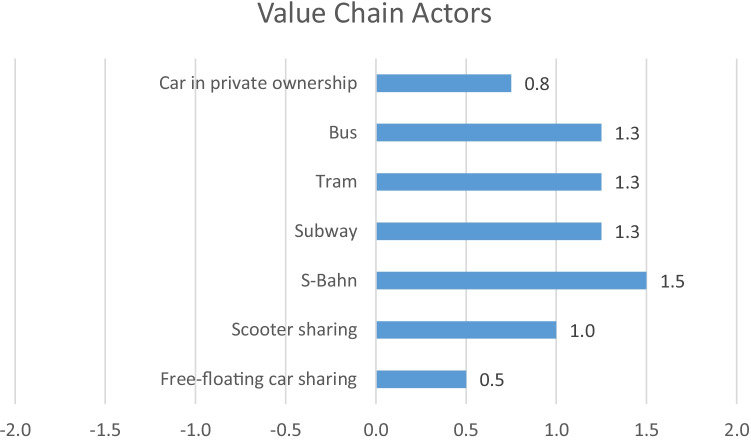
Aggregated results for the stakeholder group Value Chain Actors

**Fig. 9:**
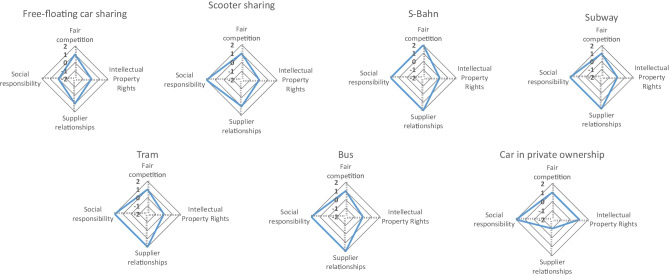
Detailed results for the stakeholder group Value Chain Actors

In Fig. 8, the aggregated results for the stakeholder group Value Chain Actors are presented, and in Fig. 9, the detailed results are shown as separate radar charts for each mobility service. In this category, all four analysed indicators are qualitative. Originally, a fifth aspect was planned to evaluate, namely the percentage of audited suppliers. For this indicator, however, it was not possible to get the necessary information for the evaluation of all mobility services, which is why this indicator was excluded from the analysis.

Regarding fair competition, all analysed mobility services, including the car in private ownership, show slightly above average results with + 1, except the S-Bahn, which receives a score of + 2. Concerning the respect of intellectual property rights, all analysed mobility services obtain an average score (0) and only the car in private ownership receives a slightly above average score of + 1. The indicator evaluating supplier relationships shows the greatest variety of results in this stakeholder category. Whereas free-floating car sharing and e-scooter sharing both show slightly above average results with + 1, the S-Bahn, subway, tram and bus reveal clearly above average results with + 2. The car in private ownership, though, shows a score of − 1. In contrast to that, the car receives a + 2 for promoting social responsibility, just like the bus, tram, subway, S-Bahn and e-scooter sharing. Free-floating car sharing receives a medium score for promoting social responsibility.

Generally, considering the social impacts for value chain actors, all mobility services have advantages over the car; however, all still have potential for improvement.

**Fig. 10:**
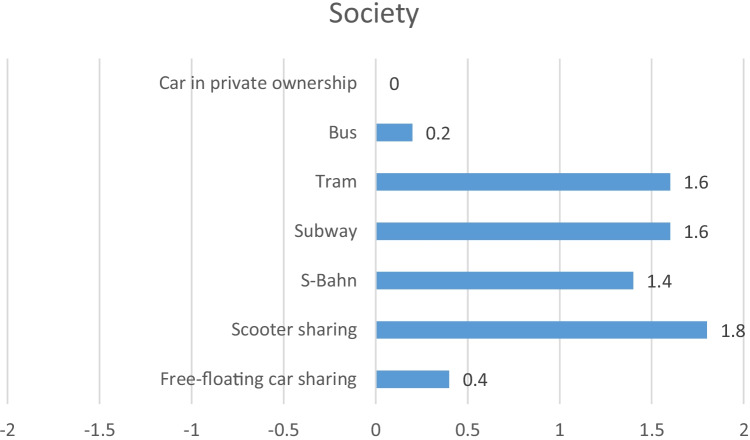
Aggregated results for the stakeholder group Society

**Fig. 11:**
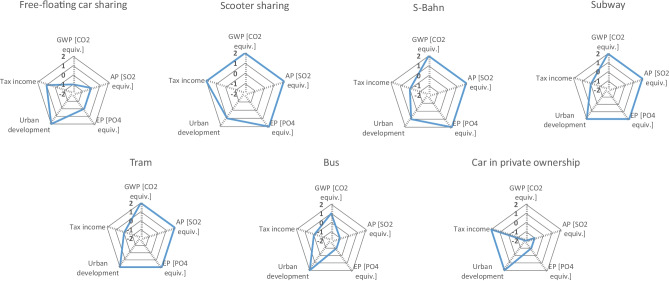
Detailed results for the stakeholder group Society

Finally, in Fig. 10, the aggregated results, and in Fig. 11, the detailed results for the stakeholder group Society are presented. All indicators in this stakeholder group are quantitative, except the indicator evaluating urban development. Having a closer look at the different mobility services, it is noticeable that the health indicators evaluating global warming potential (GWP), acidification potential (AP) and eutrophication potential (EP) receive best possible scores for the rail-bound services (S-Bahn, subway, tram). Free-floating car sharing, e-scooter sharing, the bus and car in private ownership, however, receive medium, below average or even clearly below average scores. Regarding contribution to urban development, though, all analysed mobility services, including the car in private ownership, show best possible results, except e-scooter sharing and the S-Bahn, which reveal a score of + 1. Regarding tax income, free-floating car sharing reveals a score of + 1, e-scooter sharing and the car in private ownership display a score of + 2 and S-Bahn, subway, tram and bus all show medium results.

Summarising the stakeholder category Society, it can be stated that the car, free-floating car sharing and the bus have fewer advantages for the society than other mobility services, especially considering the health indicators.

## Discussion, limitations and further research

### Discussion of case study results

This S-LCA case study provides a holistic assessment of the use phase of mobility services, taking into account five stakeholder categories and their respective social impacts. For the stakeholder group Local Community, the negative impacts related to air pollution caused by cars and buses as well as car sharing cars are a very obvious result and a well-known problem (Miller et al. 2016; Bandeira et al. 2018; Kawakubo et al. 2018; Zope et al. 2019). The results of this study underline the positive impacts of rail-bound services regarding air pollution. As the e-scooters are purely electric, it is not a surprising result that the e-scooter sharing service also shows positive impacts regarding air quality and noise pollution. Contrary, the tram reveals negative impacts regarding the noise index, which is due to the fact that the tram is running above ground and through densely populated areas.

A rather unexpected result is the negative impact of the car sharing service regarding job creation (− 2). This can be explained by the merger of DriveNow by the BMW Group and Car2Go by Daimler, which resulted in the analysed service ShareNow in February 2019. Due to the merger, many job positions existed double within the new joint venture, which is why the workforce was reduced after the merger. The indicator job creation only measures additional created jobs and therefore only shows positive results when additional jobs are created. In the case of the German railway company Deutsche Bahn, which operates the S-Bahn, or the BVG (Berlin public transportation company), which operates subway, tram and bus, a lot of people are employed and both companies are major employer in the city of Berlin. The indicator, however, does not reflect the number of existing jobs. Therefore, the indicator could be extended by this aspect. In addition, it would be desirable to be able to differentiate between job creation in general and local job creation for a better distinction between city and country level. As previously stated, it was not possible to collect the necessary data for a more detailed differentiation, which is why the indicator local job creation was not included and only hires for the whole company at country level could be analysed. Nevertheless, this is an important aspect and in case of data availability, it should definitely be included in the research.

Regarding space occupancy, it should be highlighted that free-floating car sharing reveals an advantage over the car in private ownership. By relating the occupied space to passenger kilometres (infrastructure efficiency), S-Bahn and subway show better results than the tram or the bus. Overall, the e-scooter sharing service seems to occupy the least space. This result is based on the assumption that the e-scooter sharing service only occupies space for the scooters themselves, not taking into account the corresponding infrastructure. In reality, though, the e-scooters use the same infrastructure as bikes or pedestrians and sometimes even the same infrastructure as cars. This leads to an allocation problem. For simplification, the infrastructure space occupancy by the e-scooter sharing service was not considered. The question, however, remains how to appropriately allocate the common infrastructure of pedestrians, bikes and e-scooters. The same problem occurs for the common infrastructure of cars and buses. Here, the problem could be solved by using passenger kilometre as the basis for the allocation. This, however, is not possible in the case of the e-scooters, as pedestrians and bikes are involved in the equation, for which passenger kilometres could not realistically be assumed.

As mentioned, the indicator evaluating green and open space per capita is the same for all analysed transportation options with a medium score for the city of Berlin, which is why this indicator was not included in the radar charts. Nevertheless, this indicator is considered to be relevant, especially when comparing results from different cities or the same city before and after the introduction of different mobility services.

When analysing the overall results for the stakeholder group Consumer, the negative score regarding safety for the e-scooters stands out. This result underlines the high accident rate of e-scooters (Sikka et al. 2019; Trivedi et al. 2019; Alwani et al. 2020). Contrary to the low safety of e-scooters, S-Bahn, subway and tram show very low accident rates per passenger kilometre. The car in private ownership only reveals a medium score regarding safety; nevertheless, it is the only transportation option in this stakeholder category that does not show any negative scores. This also explains why the car in private ownership is perceived as very convenient and difficult to replace by other means of transportation (Hülsmann et al. 2018; Liao et al. 2020).

For the stakeholder group Worker, it is noticeable that all transportation options show the best possible score regarding child labour and forced labour. As the study is conducted in Germany, Berlin, German law regarding these two aspects applies and is enforced. No violation concerning child labour and forced labour was detected during the study, which results in the best possible score for all services. The indicators analysing child labour and forced labour might not have the highest importance for this study, as the risk of violation in Germany is very low. Nevertheless, the indicators analysing these two aspects are considered to be highly relevant and are included in the study for comparability with other cities and countries.

For the other indicators, the public transportation services (S-Bahn, subway, tram and bus) reveal advantages over car sharing, e-scooter sharing and the car in private ownership. This can be explained by the fact that both the German railway company (Deutsche Bahn) and the Berlin public transportation company (BVG) are large companies with labour unions, codes of conduct and internal policies regarding freedom of association and work-life balance. The smaller companies that are behind the other analysed transportation options do not offer the same benefits for their workers. The same aspect also holds for the stakeholder group Value Chain Actors. The larger companies that are behind the services S-Bahn, subway, tram and bus show better results than the other transportation options as there is a code of conduct as well as internal trainings regarding the treatment of suppliers. This does not exist for employees working for the analysed companies offering car sharing, e-scooter sharing or car service stations. In the initial set of indicators, the percentage of audited suppliers is included. As mentioned, due to a lack of data, this aspect could not be evaluated, which is why it is not included in the study.

For the stakeholder category Society, the rail bound services and the e-scooter service show a clear advantage over the car, free-floating car sharing and the bus regarding the health indicators (GWP, AP, EP). This result is not surprising and underlines again the advantage of S-Bahn, subway, tram and e-scooter concerning public health. For the indicator measuring the contribution to urban development plans, the engagement of the different mobility services with the city of Berlin is analysed. Although the e-scooter sharing service only exists since a few years, the results regarding urban development are the same as for the S-Bahn Berlin, which has a long-standing cooperation with the city of Berlin. Therefore, the results can give first impressions, though it is questionable whether the indicator appropriately reflects the contribution to city development.

### Limitations and further research needs

A limitation of the study is the focus on the use phase. Although the use phase is a crucial phase when analysing mobility services, an extension of the study to other life cycle stages would be beneficial. Therefore, an extension of the study to other life cycle stages or even the entire life cycle is regarded as an area of further research need.

To a large extent, the qualitative indicators are based on interviews with employees of the respective mobility services (see Table 1). Although it can be assumed that all interviewees answered to the best of their knowledge, only a limited number of interviews were conducted and, theoretically, it could be that other departments of the analysed companies for example do not follow the code of conduct and violate internal rules without knowledge of the interviewees. Therefore, absolute reliability of the results that are based on interviews cannot be stated. One reason for the limited number of interviews is that people with thorough knowledge of the company in question were needed that have also experience in the field of sustainable mobility. Therefore, only a limited number of possible interviewees were available. In addition, the interviews were conducted during the first waves of the COVID-19 pandemic. Shared mobility operators (public transportation providers as well as car sharing/scooter sharing services) were having a hard time due to contact restrictions and hygiene requirements. This made it difficult to find experts that have time for an interview.

Another limitation is data availability for the quantitative indicators. As already mentioned, for three quantitative indicators, namely local job creation, number of consumer complaints and percentage of audited suppliers, insufficient data was available which is why these three indicators could not be included in the study. For other indicators, data was available, however, not in the desirable quality. This was the case for the indicators noise pollution and space occupancy. The data set used for noise pollution of the S-Bahn includes noise pollution of all trains operated by the German railway company, which also includes regional and long-distance trains that are running on the same railways. Although areas were excluded where there is no S-Bahn traffic, the noise pollution of the S-Bahn might in reality be lower than assumed in this study due to the lack of specific data. In order to model the space occupied by different mobility services, various data sets were used. The data set for parking spaces is based on open source data, and therefore, missing data cannot be excluded with certainty. Moreover, for the space occupied by railway tracks, only data about the length of the railway tracks is available. For the calculation of the occupied space, the width had to be estimated.

As previously stated, the e-scooters use the same infrastructure as bicycles or pedestrians and sometimes even the same infrastructure as cars, which leads to an allocation problem. How to appropriately allocate the common infrastructure, namely bike lanes, pavements and streets, is considered to be an important area of further research.

Another possible area of further research relates to the indicators job creation and contribution to urban development. This case study showed that both indicators leave out relevant aspects, specifically the total number of employed persons and the years of cooperation with the city. How to include these aspects and better reflect the reality could be further examined.

Finally, a fundamental area of further research is the application of the stated set of indicators to other cities, especially to other types of cities and different geographical areas. This case study gives results for the city of Berlin in Germany, outlines a procedure and gives insights regarding limitations. Validating the set of indicators and evaluating the reference scales in other case studies is considered to be a crucial area of further research.

## Conclusion and recommendations

This S-LCA case study provides an assessment of social impacts related to different mobility services in Berlin, evaluating social impacts of the use phase for free-floating car sharing, e-scooter sharing, S-Bahn, subway, tram, bus and the car in private ownership. For the analysis, five stakeholder groups that are outlined in the S-LCA Guidelines (UNEP 2020) were taken into account: Local Community, Consumer, Worker, Value Chain Actors and Society. For a detailed analysis of all relevant aspects, the set of indicators and corresponding reference scales as proposed by Gompf et al. (2020) were used for the evaluation. In total, 36 indicators were analysed, out of which 22 are quantitative and 14 are qualitative. For data collection, several different data sources were used, including publicly available data e.g. from statistics as well as own data from interviews. Partly, the results of the case study are as expected, for example regarding impacts on air quality. Partly however, the results are specific for the analysed mobility services in Berlin and therefore give new insights and reveal new aspects, as for example in the case of job creation for the local community.

The main challenge of this case study was data availability and data quality, which is why assumptions and simplifications were used for some indicators, especially regarding space occupancy and the allocation of common infrastructure. A major limitation of the study is that the results of the qualitative indicators are based on a limited number of interviews and absolute reliability of the results cannot be guaranteed. For an improvement of the results and to further validate the chosen set of indicators with the corresponding reference scales, the study should be expanded to other cities with different mobility services in other geographical regions. Evaluating mobility services in other S-LCA case studies for validation and verification of the results is regarded as an essential area of further research.

Generally, this S-LCA case study provides a holistic assessment of the use phase and a complete overview considering five stakeholder categories and their respective social impacts. As the assessment of the use phase has been underrepresented in previous S-LCA case studies, this research contributes to close the gap regarding S-LCA studies that focus on the use phase. Especially for mobility services, the analysis of the use phase is important. Thus, the results of this study can help researchers and practitioners in the field of urban mobility assessment as it systematically analyses social sustainability aspects of mobility services and outlines a procedure for further research. That way, this case study contributes to answer the overlying question whether mobility services can improve quality of life in cities.

## Supplementary information

Below is the link to the electronic supplementary material.

Supplementary file1 (DOCX 70 KB)

## Data Availability

All data generated or analysed during this study are included in this published article and its supplementary information files.

## References

[CR1] Alwani M, Jones AJ, Sandelski M (2020). Facing facts: facial injuries from stand-up electric scooters. Cureus.

[CR2] Bandeira RAM, D’Agosto MA, Ribeiro SK (2018). A fuzzy multi-criteria model for evaluating sustainable urban freight transportation operations. J Clean Prod.

[CR3] Benoit-Norris C, Cavan DA, Norris G (2012). Identifying social impacts in product supply chains: overview and application of the social hotspot database. Sustainability.

[CR4] Berliner Verkehrsbetriebe (BVG) (2019) Nachhaltige Unternehmensentwicklung Fortschrittsmitteilung 2018 / 2019 zum UN Global Compact Berliner Verkehrsbetriebe (BVG)

[CR5] Bilali A, Rathore MAA, Fastenrath U, Bogenberger K (2020) An analytical model to evaluate traffic impacts of on-demand ride pooling. In: 2020 IEEE 23rd International Conference on Intelligent Transportation Systems, ITSC 2020

[CR6] Chhipi-Shrestha GK, Hewage K, Sadiq R (2014). ‘Socializing’ sustainability : a critical review on current development status of social life cycle impact assessment method. Clean Technol Environ Policy.

[CR7] Civity (2021) E-Scooter in Deutschland: Ein datenbasierter Debattenbeitrag. https://scooters.civity.de/

[CR8] Curran MA (1996). Environmental life-cycle assessment. Int J Life Cycle Assess.

[CR9] Di Cesare S, Silveri F, Sala S (2018) Positive impacts in social life cycle assessment : state of the art and the way forward. 406–421. 10.1007/s11367-016-1169-7

[CR10] do Carmo BBT, de Oliveira Castro G, Gonçalo TEE, Ugaya CML (2021) Participatory approach for pertinent impact subcategory identification: local community. Int J Life Cycle Assess 26:950–962.10.1007/s11367-021-01892-3

[CR11] Dubois-Iorgulescu A-M, Saraiva AKE, Valle R, Rodrigues LM (2016) How to define the system in social life cycle assessments ? A critical review of the state of the art and identification of needed developments. Int J Life Cycle Assess. 10.1007/s11367-016-1181-y

[CR12] Finkbeiner M, Schau EM, Lehmann A, Traverso M (2010). Towards Life Cycle Sustainability Assessment Sustain.

[CR13] Fontes J, Carmen A, Saling P et al (2016) Handbook for product social impact assessment. https://www.researchgate.net/publication/312802992_Handbook_for_Product_Social_Impact_Assessment_30. Accessed 30 Dec 2019

[CR14] Gerike R, Hubrich S, Ließke F et al (2020) Sonderauswertung zum Forschungsprojekt “Mobilität in Städten – SrV 2018” Stadtgruppe: SrV-Städtepegel

[CR15] Goedkoop MJ, Indrane D, de Beer IM (2018) Handbook for product social impact assessment. https://product-social-impact-assessment.com/. Accessed 30 Dec 2019

[CR16] Gompf K, Traverso M, Hetterich J (2020). Towards social life cycle assessment of mobility services: systematic literature review and the way forward. Int J Life Cycle Assess.

[CR17] Gompf K, Traverso M, Hetterich J (2021). Using analytical hierarchy process (AHP) to introduce weights to social life cycle assessment of mobility services. Sustain.

[CR18] Gould E, Wehrmeyer W, Leach M, Electric S (2015). Transition pathways of e-mobility services. Trans Ecol Environ.

[CR19] Gross M (2019). The future is urbanised. Curr Biol.

[CR20] Hietanen S (2014). Mobility as a Service – the new transport model? ITS & Transport Management Supplement. Eurotransport.

[CR21] Huarachi D, Moro C, Neves F (2020). Past and future of social life cycle assessment : historical evolution and research trends. J Clean Prod.

[CR22] Huertas-Valdivia I, Ferrari AM, Settembre-Blundo D, García-Muiña FE (2020). Social life-cycle assessment: a review by bibliometric analysis. Sustain.

[CR23] Hülsmann F, Wiepking J, Zimmer W (2018) share – Wissenschaftliche Begleitforschung zu car2go mit batterieelektrischen und konventionellen Fahrzeugen. https://www.oeko.de/fileadmin/oekodoc/share-Wissenschaftliche-Begleitforschung-zu-car2go-mit-batterieelektrischen-und-konventionellen-Fahrzeugen.pdf

[CR24] IPCC (2014) Climate Change 2014: mitigation of climate change. Contribution of Working Group III to the Fifth Assessment Report of the Intergovernmental Panel on Climate Change

[CR25] ISO 14040 (2006) Environmental management - life cycle assessment - principles and framework

[CR26] Jittrapirom P, Caiati V, Feneri A-M et al (2017) Mobility as a service : a critical review of definitions, assessments of schemes and key challenges. Urban Plan 2:13–25. 10.17645/up.v2i2.931

[CR27] Kawakubo S, Murakami S, Ikaga T, Asami Y (2018). Sustainability assessment of cities: SDGs and GHG emissions. Build Res Inf.

[CR28] Kühnen M, Hahn R (2017). Indicators in social life cycle assessment: a review of frameworks, theories, and empirical experience. J Ind Ecol.

[CR29] Liao F, Molin E, Timmermans H, van Wee B (2020). Carsharing: the impact of system characteristics on its potential to replace private car trips and reduce car ownership.

[CR30] Miller P, de Barros AG, Kattan L, Wirasinghe SC (2016). Analyzing the sustainability performance of public transit. Transp Res Part D Transp Environ.

[CR31] Parent J, Cucuzzella C, Revéret J (2010) Impact assessment in SLCA : sorting the sLCIA methods according to their outcomes. Int J Life Cycle Assess c:164–171. 10.1007/s11367-009-0146-9

[CR32] Petti L, Serreli M, Cesare S Di (2016) Systematic literature review in social life cycle assessment. Int J Life Cycle Assess 422–431.10.1007/s11367-016-1135-4

[CR33] Russo Garrido S, Parent J, Beaulieu L, Revéret J (2016). A literature review of type I SLCA — making the logic underlying methodological choices explicit. Int J Life Cycle Assess.

[CR34] S-Bahn Berlin (2021) S-Bahn Berlin: Auf einen Blick - Zahlen und Fakten. https://sbahn.berlin/das-unternehmen/unternehmensprofil/auf-einen-blick-zahlen-und-fakten/

[CR35] ShareNow (2021) ShareNow: Facts & Figures. https://www.share-now.com/de/en/corona-carsharing-facts-figures/

[CR36] Siebert A, Keeffe SO, Bezama A, Zeug W (2018) How not to compare apples and oranges : generate context-speci fi c performance reference points for a social life cycle assessment model. J Clean Prod 19810.1016/j.jclepro.2018.06.298

[CR37] Sikka N, Vila C, Stratton M (2019). Sharing the sidewalk: a case of E-scooter related pedestrian injury. Am J Emerg Med.

[CR38] Sureau S, Neugebauer S, Achten W (2019) Different paths in social life cycle impact assessment ( S-LCIA ) — a classification of type II impact pathway approaches. Int J Life Cycle Assess

[CR39] Tokede O, Traverso M (2020) Implementing the guidelines for social life cycle assessment : past , present , and future. Int J Life Cycle Assess 1–11

[CR40] Trivedi B, Kesterke MJ, Bhattacharjee R (2019). Craniofacial injuries seen with the introduction of bicycle-share electric scooters in an urban setting. J Oral Maxillofac Surg.

[CR41] UNEP/SETAC (2009) Guidelines for social life cycle assessment of products. http://wedocs.unep.org/handle/20.500.11822/7912. Accessed 30 Dec 2019

[CR42] UNEP/SETAC (2013) The methodological sheets for sub-categories in social life cycle assessment (S-LCA). https://www.lifecycleinitiative.org/wp-content/uploads/2013/11/S-LCA_methodological_sheets_11.11.13.pdf. Accessed 30 Dec 2019

[CR43] UNEP (2020) Guidelines for social life cycle assessment of products and organizations. In: United Nations Environ. Program. https://www.lifecycleinitiative.org/library/guidelines-for-social-life-cycle-assessment-of-products-and-organisations-2020/. Accessed 18 Jan 2021

[CR44] Weidema BP (2018). The social footprint—a practical approach to comprehensive and consistent social LCA. Int J Life Cycle Assess.

[CR45] Zope R, Vasudevan N, Arkatkar SS, Joshi G (2019). Benchmarking: a tool for evaluation and monitoring sustainability of urban transport system in metropolitan cities of India. Sustain Cities Soc.

