# Supplementary material for: Applying social life cycle assessment to evaluate the use phase of mobility services: a case study in Berlin
Source: Int J Life Cycle Assess. 2022 Apr 28;27(4):603–22. doi: 10.1007/s11367-022-02051-y (PMC9046070; doi:10.1007/s11367-022-02051-y)
Supplement: Supplementary file 1 — Supplementary file1 (DOCX 70 KB) [file 11367_2022_2051_MOESM1_ESM.docx]

**Supporting Information**

**for**

Applying social life cycle assessment to evaluate the use phase of mobility services: a case study in Berlin

Katharina Gompf ^1,2^, Marzia Traverso^1^, Jörg Hetterich^2^

^1^ *Institute of Sustainability in Civil Engineering, RWTH Aachen University, Aachen, Germany*

^2^*BMW Group, Knorrstraße 147, 80788 Munich, Germany*

*Corresponding author: Katharina Gompf,* [*Katharina.Gompf@bmw.de*](mailto:Katharina.Gompf@bmw.de)*, Mobil: +49-151-60177352*

***Keywords:*** *Mobility Services, Social life cycle assessment, S-LCA, Use phase, case study, social sustainability indicators*

**S1** Inventory data for all quantitative indicators

**S2** Inventory data for all qualitative indicators

**S3** Questionnaire

Topic - Customer concerns

| **Community engagement** | Please assess:  **The community, residents and citizens have the opportunity to comment on or vote on your company's projects and make their concerns heard.**   \| does not apply at all \| does rather not apply \| partially applies \| largely applies \| fully applies \| \| --- \| --- \| --- \| --- \| --- \| \|  \|  \|  \|  \|  \|   How do you justify your decision? |
| --- | --- | --- | --- | --- | --- | --- | --- | --- | --- | --- | --- |
|  | **Please select the options that apply to your company**   \|  \| There is a method of addressing requests and complaints from residents or citizens' initiatives. \| \| --- \| --- \| \|  \| There are rules and timelines for how these requests and complaints must be handled - to ensure they are discussed. \| \|  \| There is an open dialog with citizen representatives, customer representatives, etc., whose opinions are taken into account in management decisions. \| \|  \| There are rules that the society/community must be involved if your company's decisions or changes have a big impact on them. *(The bus line is rerouted or the frequency of the subway is significantly reduced, etc.)* \| |
| **City of Berlin** | Please assess:  **Your company is in constant exchange with the city of Berlin in order to make a positive contribution to urban development.**   \| does not apply at all \| does rather not apply \| partially applies \| largely applies \| fully applies \| \| --- \| --- \| --- \| --- \| --- \| \|  \|  \|  \|  \|  \|   How do you justify your decision? |
| **Inclusive design** | To what extent do you find that all stations and vehicles are accessible to all user groups (especially with regard to people with disabilities and poorer people)?  How do you justify your decision? |
|  | **Do you aim (in the future) to offer your vehicles to as many people as possible, including older or poorer people?**   - If so, what does this look like? |
|  | **What are the discounts for disabled, elderly or poorer people?** |
| **Data privacy** | **How does your company benefit from selling/sharing your customer data?** |
|  | **Does your company have rules in place to protect customer data?** |
|  | **Is there a possibility for customers to**   \|  \| View their data? \| \| --- \| --- \| \|  \| To prohibit the company from using their data? \| \|  \| Correct incorrect data? \| \|  \| Delete the data? \| \|  \| Restrict the processing of data? \| \|  \| Transfer the data? \| |
|  | **Is there a device (website, contact, etc.) where privacy complaints can be filed?** |
|  | **Are the rules and liabilities for data protection publicly viewable?** |

Topic - Employee

| **Remuneration** | **Have there ever been complaints that workers can't cover their living expenses with their wages?** |
| --- | --- |
|  | **What additional benefits are offered equally to all employees?**   \|  \| Company pension plan \|  \| ___________________________ \| \| --- \| --- \| --- \| --- \| \|  \| Company health insurance \|  \| ___________________________ \| \|  \| Transportation discount (Job-Ticket) \|  \| ­­­­­___________________________ \| |
|  | **Are there any known cases where your company has paid employees late or has generally fallen behind in payments?** |
| **Work-Life Balance** | **To what extent can employees freely organize their working hours?**  (e.g. flexible working hours, core hours, shift work) |
|  | **Can employees cut back or add back their hours if needed?** |
|  | **Can employees take their legally available parental leave at will?**   - How many of the workers accept the offer? (approximate estimation) |
|  | **What is the maximum number of hours worked per week?**  (Overtime is excluded)  Have there been situations in the past where a shift employee has to work more than 48 hours per week? |
|  | **Does your company advertise work-life balance?**   - **does your company offer additional benefits for a good work-life balance?**  (e.g. sports offers, coaching, etc.) |
|  | **Are your company's activities to promote work-life balance publicly visible?** |
| **Equal treatment** | **Does your company have a department, company policy, equality officer(s), or similar to ensure equal treatment?**  (Gender, age, sexual orientation, disability, ethnicity, nationality, religion, etc.) |
|  | **Is equal treatment considered a major focus of top management?** |
|  | **Does your company have preventive measures in place to protect against discrimination?**   - If yes, which ones? |
|  | **Are you aware of any instances where there have been complaints of disadvantage or discrimination?**   - If yes, were measures taken as a result? |
|  | **Does your company report publicly on its equal treatment policy?** |
| **Freedom of association** | **Is there a general regulation/company policy that gives employees the freedom to join labour unions or private organizations?** |
|  | **Can you estimate approximately how many employees in your company have joined a union?** |
|  | **Does your company have a workers' council that represents the interests of the employees to the Board of Management?**   - If not, is a foundation planned? - If not, would you find the introduction of a workers‘ council critical? - If so, what is the cooperation between the workers' council and the employer like? Is the workers' council accepted? |
|  | **Does your company accept its workers' council and unions as negotiating partners in collective bargaining, etc.?** |
|  | **Are you aware of cases where employees have been directly or indirectly prohibited from joining a labour union or forming a workers council/standing for election?**   - If so, was any action taken as a result? |

Topic - Business partner

| **Fair trading relationships** | **Does your company have a policy of complying with the rules of fair competition? (Antitrust law, no collusion with competitors, etc.)**   - How is it ensured that these rules are followed? | | | | | |
| --- | --- | --- | --- | --- | --- | --- |
|  | **(How) Are employees trained on compliance with fair competition and antitrust rules?** | | | | | |
|  | **When selecting (new) business partners, suppliers, etc., is it ensured that they also comply with national and international laws on fair competition?** | | | | | |
|  | **Are business partners, suppliers, etc. actively encouraged by your company to likewise comply with the rules on fair competition, for their own suppliers in turn?** | | | | | |
|  | **Are you aware of any violations of fair competition rules by your company?** | | | | | |
| **Intellectual property** | Please assess:  **At your company, intellectual property protection (of business partners, suppliers, etc.) is emphasized throughout the company and corresponding laws are followed.**   \| does not apply at all \| does rather not apply \| partially applies \| largely applies \| fully applies \| \| --- \| --- \| --- \| --- \| --- \| \|  \|  \|  \|  \|  \|   How do you justify your decision? | | | | | |
|  | **Does your company have a policy of handling the intellectual property of third parties responsibly and within the legal framework?**   - How to ensure that this principle is respected? | | | | | |
|  | **Is there a continuous improvement process (PDCA process) to comply with the intellectual property rights of third parties?** | | | | | |
|  | **Are you aware of any cases in which your company has infringed the intellectual property rights of others?**   - If so, what was the response? | | | | | |
| **Promotion of social sustainability** | **Does your company have a code of conduct or similar that also takes into account the (human) rights of employees of business partners, etc.?**   - How is it ensured that this code is followed? | | | | | |
|  | **Is your company a member of an initiative that supports social responsibility along the supply chain?** (e.g. Global Social Compliance Programme - GSCP - industry initiative to improve sustainability standards in the supply chain; Fair Labor Association FLA) | | | | | |
|  | **Are clauses on ethical, social and gender-neutral responsibility added to contracts with business partners, suppliers, etc.? Are clauses on ethical, social, environmental and gender-neutral responsibility added?** | | | | | |
|  | **(How) Does your company support the business partners, suppliers, etc. to raise awareness of social responsibility? (e.g. by training)** | | | | | |
|  | **Are business partners, suppliers, etc. actively encouraged by your company to in turn promote social responsibility to their own suppliers as well?** | | | | | |
|  | **Are business partners, suppliers, etc. audited internally regarding their social responsibility?**   - If yes, what percentage of your business partners have been audited in 2019? | | | | | |
| **Supplier Relationship** | | does not apply at all | does rather not apply | partially applies | largely applies | fully applies |
| **Your company always treats its suppliers and business partners respectfully and well.** | |  |  |  |  |  |
| **Your company always gives its suppliers, business partners sufficient processing time.** | |  |  |  |  |  |
| **Your company always ensures adequate utilization of its suppliers and business partners.** | |  |  |  |  |  |
| **Your company always pays its suppliers and business partners on time.** | |  |  |  |  |  |
| **Communication between your company and its suppliers and business partners is always at equal level.** | |  |  |  |  |  |
